# Estimated GFR Accuracy When Cystatin C– and Creatinine-Based Estimates Are Discrepant in Older Adults

**DOI:** 10.1016/j.xkme.2023.100628

**Published:** 2023-03-13

**Authors:** O. Alison Potok, Dena E. Rifkin, Joachim H. Ix, Michael G. Shlipak, Anita Satish, Alice Schneider, Nina Mielke, Elke Schaeffner, Natalie Ebert

**Affiliations:** 1 Division of Nephrology-Hypertension, University of California San Diego, San Diego, CA; 2 Veterans Affairs San Diego Healthcare System, San Diego, CA; 3 Kidney Health Research Collaborative, University of California, San Francisco, CA; 4 Department of Medicine, University of California San Francisco, San Francisco, CA; 5 University of California San Diego School of Medicine, San Diego, CA; 6 Charité–Universitätsmedizin Berlin, Institute of Public Health, Berlin, Germany

**Keywords:** Estimated glomerular filtration rate (eGFR), measured GFR, iohexol, cystatin C, creatinine, accuracy, bias

## Abstract

**Rationale & Objective:**

Serum creatinine and cystatin C are used to estimate glomerular filtration rate, but creatinine-based estimated glomerular filtration rate (eGFRcr), cystatin C–based estimated glomerular filtration rate (eGFRcys), and combined creatinine- and cystatin C–based estimated glomerular filtration rate (eGFRcr-cys) are often divergent, particularly in older adults. We investigated which estimated glomerular filtration rate (eGFR) was more accurate and less biased compared with measured glomerular filtration rate (mGFR).

**Study Design:**

A diagnostic test study from the Berlin Initiative Study.

**Setting & Participants:**

The study population included 657 individuals aged 70 years or older with iohexol plasma clearance (mGFR) and serum creatinine and cystatin C measurements: 567 community-dwelling participants and 90 with a serum creatinine of ≥1.5 mg/dL.

**Tests Compared:**

We defined 3 groups on the basis of the difference eGFRcys − eGFRcr: whether < −5 mL/min/1.73 m^2^ (lower eGFRcys), within 5 mL/min/1.73 m^2^ (reference), or **≥** 5 mL/min/1.73 m^2^ (lower eGFRcr). eGFRcr, eGFRcys, and eGFRcr-cys were compared to mGFR to assess bias and accuracy.

**Outcome:**

Median bias (eGFR minus mGFR) with 95% CIs and accuracy (percentage of eGFR values within ±30% of mGFR).

**Results:**

The mean ± standard deviation age was 78 ± 6 years; the mean eGFRcys, eGFRcr, and eGFRcr-cys were 59 ± 23, 64 ± 20, and 61 ± 22 mL/min/1.73 m^2^, respectively, and the mean mGFR was 56 ± 19 mL/min. Half of the participants were in the lower eGFRcys group (n=337, 51%). Among them, the median bias for eGFRcys was the lowest (median bias, −2.7; 95% CI, −3.8 to −1.9) compared with the other eGFR equations. Conversely, in the lower eGFRcr group (n=121, 18%), the median bias for eGFRcr was the lowest compared with those for eGFRcys and eGFRcr-cys (2.9; [95% CI, 0.9-4.8] vs 13.8 [95% CI, 11.4-15.6] and 9.5 [95% CI, 7.7-11.0], respectively). Accuracy (percentage of eGFR values within ±30% of mGFR) was 93% for eGFRcr in the lower eGFRcr group and 92% for eGFRcys and 94% for eGFRcr-cys in the lower eGFRcys group.

**Limitations:**

Untested generalizability in younger populations.

**Conclusions:**

Among older adults, the lower eGFR between eGFRcys and eGFRcr was a more accurate and less biased estimate of mGFR when comparing the groups.

## Graphical abstract

**Figure:**
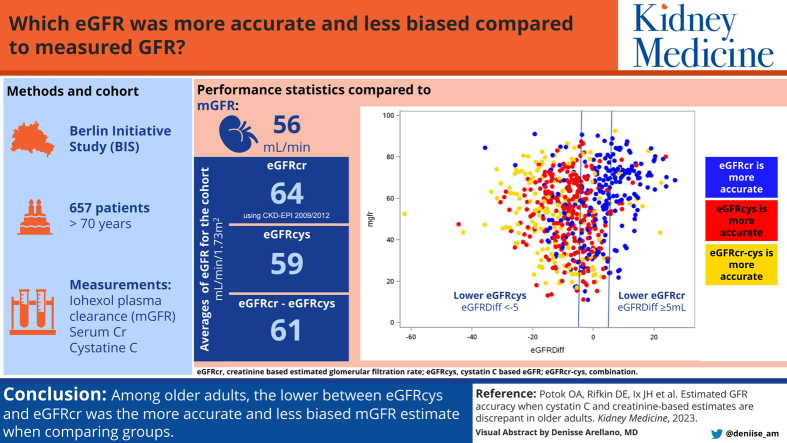

**Plain-Language Summary**

Better assessment of kidney function in older persons is needed to improve clinical care and drug dosing. Kidney function is calculated using serum creatinine, cystatin C, or both (creatinine-based estimated glomerular filtration rate [eGFR] vs cystatin C–based eGFR vs combined). This study shows that the lower eGFR between creatinine-based eGFR and cystatin C–based eGFR is the more accurate estimate of measured glomerular filtration rate in older adults. The findings may have important clinical implications for kidney disease diagnosis, prognosis, and drug dosing in older patients.

Accurate estimation of glomerular filtration rate (GFR) is important for the diagnosis of kidney disease, risk stratification, and medication dosing. The gold standard to assess kidney function is the invasive measurement of GFR using an exogenous marker, such as iohexol or iothalamate.^1^^,^^2^ However, measured glomerular filtration rate (mGFR) is not widely used or available in clinical practice because it entails standardized protocols, extended sampling time and repeated blood measurements, and requirement of specialized laboratories. A widespread alternative to measuring GFR is the use of endogenous serum biomarkers that can be used to estimate GFR. Serum creatinine has been used for decades to assess kidney function,^3^ although it is known to be influenced by many factors other than GFR, such as diet, muscle mass and activity, sex, and age.^4^, ^5^, ^6^ Cystatin C is an alternative endogenous marker that is less influenced by muscle than creatinine^7^ and has been shown to improve the overall accuracy of GFR estimation equations when used in combination with creatinine, particularly in older adults.^8^^,^^9^ Additionally, cystatin C–based estimated glomerular filtration rate (eGFRcys) has been found to be more strongly associated with cardiovascular events and mortality.^10^, ^11^, ^12^

Because cystatin C has become more widely used in clinical practice, clinicians are often faced with discrepant GFR estimates (using cystatin C, creatinine, or both combined) within the same individual.^13^^,^^14^ Wider discrepancies are particularly common in older, frail adults. In prior work,^15^^,^^16^ we have demonstrated that the degree of this discrepancy, particularly when estimated glomerular filtration rate (eGFR) by cystatin is lower than that by creatinine, is strongly associated with frailty, hospitalization rates, and mortality. Yet, when the estimates are discrepant from one another, deciding which one of the estimates is the most accurate estimate of mGFR is challenging and is of critical importance to clinical care because many medications are dosed on the basis of kidney function, and older adults are typically prescribed multiple medications concurrently, increasing the risk of drug-drug interactions and adverse drug effects.

The aim of this study was to determine whether the eGFR estimated by cystatin C, creatinine, or both combined is most accurate when the estimates are divergent in older adults. We used data from the Berlin Initiative Study (BIS),^17^ a cohort of German adults aged 70 years or older, in whom we compared cystatin C-based eGFR (eGFRcys), creatinine-based eGFR (eGFRcr), or both creatinine and cystatin C - based eGFR (eGFRcr-cys) with mGFR on the basis of iohexol plasma clearance. Our aim was to determine which eGFR had the highest accuracy and least bias relative to mGFR in the overall population as well as within the groups on the basis of the difference in eGFR by cystatin C versus creatinine (eGFRDiff = eGFRcys − eGFRcr). Based on our prior work, we hypothesized that eGFRcys would outperform eGFRcr as well as eGFRcr-cys overall and particularly when eGFRcys is lower than eGFRcr in this elderly population.

## Methods

### Study Population

BIS is a cohort study of persons aged 70 years and older living in Berlin and recruited through one of the largest German health insurance companies. Details of the enrollment have been previously described.^17^ The present study included 567 participants from the initial BIS study who had GFR measured by iohexol plasma clearance (mGFR) as well as serum creatinine and cystatin C levels. Because BIS is a community-dwelling cohort with a relatively low prevalence of kidney disease, we additionally included 90 participants with decreased kidney function from a satellite project of BIS.^18^ These elderly participants were receiving care from a nephrologist and were recruited through cooperating nephrology outpatient clinics. The inclusion criteria were an age of 70 years and above, decreased kidney function with an externally measured ambulatory serum creatinine level of at least 1.5 mg/dL, a thyroid-stimulating hormone level of >0.3 mIU/L, and no known iodine allergy. Therefore, the resulting total study population included 657 individuals. BIS was approved by the local ethics committee, and every participant gave written informed consent.^9^ All research was performed in accordance with the Declaration of Helsinki.

### Tests Compared

eGFR using various estimating equations was compared with mGFR, and performance analyses were conducted. eGFRcr and eGFRcys were calculated using the 2009^19^ and 2012^8^ CKD-EPI (Chronic Kidney Disease Epidemiology Collaboration) equations, respectively. eGFRcr-cys was calculated using the 2012 CKD-EPI equation.^8^ Serum creatinine was analyzed using the isotope dilution mass spectrometry traceable enzymatic method (CREA Plus; Roche Diagnostics) on a Roche modular analyzer P-Module. Cystatin C was measured using a particle-enhanced nephelometric assay on the BN ProSpec nephelometer (Siemens Healthcare Diagnostics).

Because the National Kidney Foundation and American Society of Nephrology Task Force’s recommendations^20^ support the use the 2021 CKD-EPI equations to estimate GFR, analyses were repeated using the 2021 CKD-EPI equations.^21^ Sensitivity analyses using the full-age spectrum (FAS) equations were performed.^22^

### Clinical Outcome

GFR was measured (mGFR) using plasma clearance over 5 hours (based on the decay of plasma concentrations over time) of exogenous iohexol. The protocol for this measurement has been described previously.^9^

### Statistical Analysis

We examined the population as a whole and then stratified it into 3 groups on the basis of the difference in the eGFR estimates: (1) those in whom eGFRDiff = eGFRcys − eGFRcr was strictly less than −5 mL/min/1.73 m^2^ (lower eGFRcys group); (2) those in whom eGFRcr and eGFRcys were within 5 mL/min/1.73 m^2^ of one another in either direction (reference group); and (3) those in whom eGFRDiff = eGFRcys − eGFRcr was ≥5 mL/min/1.73 m^2^ (lower eGFRcr group). We evaluated the participants’ baseline characteristics using mean (± standard deviation) for continuous variables and count (percentage) for categorical variables. To determine which eGFR was the most accurate estimate of mGFR (between eGFRcr, eGFRcys, and eGFRcr-cys), we calculated the median bias (ie, eGFR − mGFR) with 95% confidence intervals (CIs) and percentage of eGFR values within ±30% of mGFR (P30) accuracy with 95% confidence intervals.^23^

Statistical analyses were conducted using SAS version 9.4 and SAS Enterprise version 7.1 (SAS Institute), with *P* values of <0.05 considered statistically significant.

## Results

The baseline characteristics of the 657 study participants are summarized in Table 1. The mean (± standard deviation) age was 78 (±6) years; the mean eGFRcys, eGFRcr, and eGFRcr-cys were 59 (±23), 64 (±20), and 61 (±22) mL/min/1.73 m^2^, respectively, and the mean mGFR was 56 (±19) mL/min. The mean (± standard deviation) body mass index was 28 (±4) kg/m^2^, 58% were men, and 26% had diabetes mellitus. Half of the participants (51%) had a lower eGFRcys than eGFRcr by >5 mL/min/1.73 m^2^ (ie, the lower eGFRcys group).

**Table 1:** Baseline Characteristics of Participants, by Group Based on Which eGFR is Lower Between Cystatin C– and Creatinine-Based eGFR

	Lower eGFRcys	Reference	Lower eGFRcr	Total
	eGFRDiff < -5 mL/min/1.73 m^2^	−5 ≤ eGFRDiff <5 mL/min/1.73 m^2^	eGFRDiff ≥ 5 mL/min/1.73 m^2^	
n (%)	337 (51)	199 (30)	121 (18)	657
Baseline age (y), mean (SD)	79 (6)	78 (6)	76 (5)	78 (6)
Male, n (%)	212 (63)	110 (55)	61 (50)	383 (58)
Difference: eGFRcys − eGFRcr (mL/min/1.73 m^2^), mean (SD)	−14 (7)	−0.4 (3)	12 (5)	−5 (12)
Difference: eGFRcys − eGFRcr, (mL/min/1.73 m^2^), median (IQR)	−12.7 (−17.6 to −8.4)	−0.4 (−3.0 to 1.9)	10.4 (7.5 to 14.5)	−5.5 (−12.9 to 2.2)
Serum creatinine (mg/dL), mean (SD)	1.06 (0.40)	1.29 (0.65)	0.98 (0.37)	1.11 (0.50)
Serum cystatin C (mg/L), mean (SD)	1.41 (0.55)	1.38 (0.64)	0.93 (0.26)	1.31 (0.57)
eGFRcr (mL/min/1.73 m^2^), mean (SD)	66 (19)	57 (23)	70 (18)	64 (20)
eGFRcys (mL/min/1.73 m^2^), mean (SD)	52 (18)	56 (23)	82 (19)	59 (23)
eGFRcr-cys (mL/min/1.73 m^2^), mean (SD)	58 (19)	57 (23)	77 (20)	61 (22)
mGFR (mL/min), mean (SD)	54 (18)	51 (19)	69 (17)	56 (19)
Prevalence of an mGFR of <60 mL/min, n (%)	201 (60)	128 (64)	31 (26)	360 (55)
Urine albumin-to-creatinine ratio (mg/g), median (IQR)	14.2 (5.6 to 37.7)	11.6 (4.5 to 42.7)	7.9 (4.4 to 21.3)	11.85 (4.76 to 35.87)
BMI (kg/m^2^), mean (SD)	28 (4)	28 (4)	28 (4)	28 (4)
Diabetes mellitus, n (%)	95 (28)	51 (26)	27 (22)	173 (26)
Systolic BP (mm Hg), mean (SD)	143 (23)	143 (20)	145 (19)	143 (22)
Diastolic BP (mm Hg), mean (SD)	81 (13)	80 (13)	83 (13)	81 (13)

Abbreviations and definitions: BMI, body mass index; BP, blood pressure; eGFR, estimated glomerular filtration rate; eGFRcr, creatinine-based estimated glomerular filtration rate; eGFRcr-cys, combined creatinine- and cystatin C–based estimated glomerular filtration rate; eGFRcys, cystatin C–based estimated glomerular filtration rate; eGFRDiff = eGFRcys − eGFRcr using the CKD-EPI (Chronic Kidney Disease Epidemiology Collaboration) eGFR equations from 2012 and 2009, respectively; IQR, interquartile range; mGFR, measured glomerular filtration rate; SD, standard deviation.

**Figure 1:**
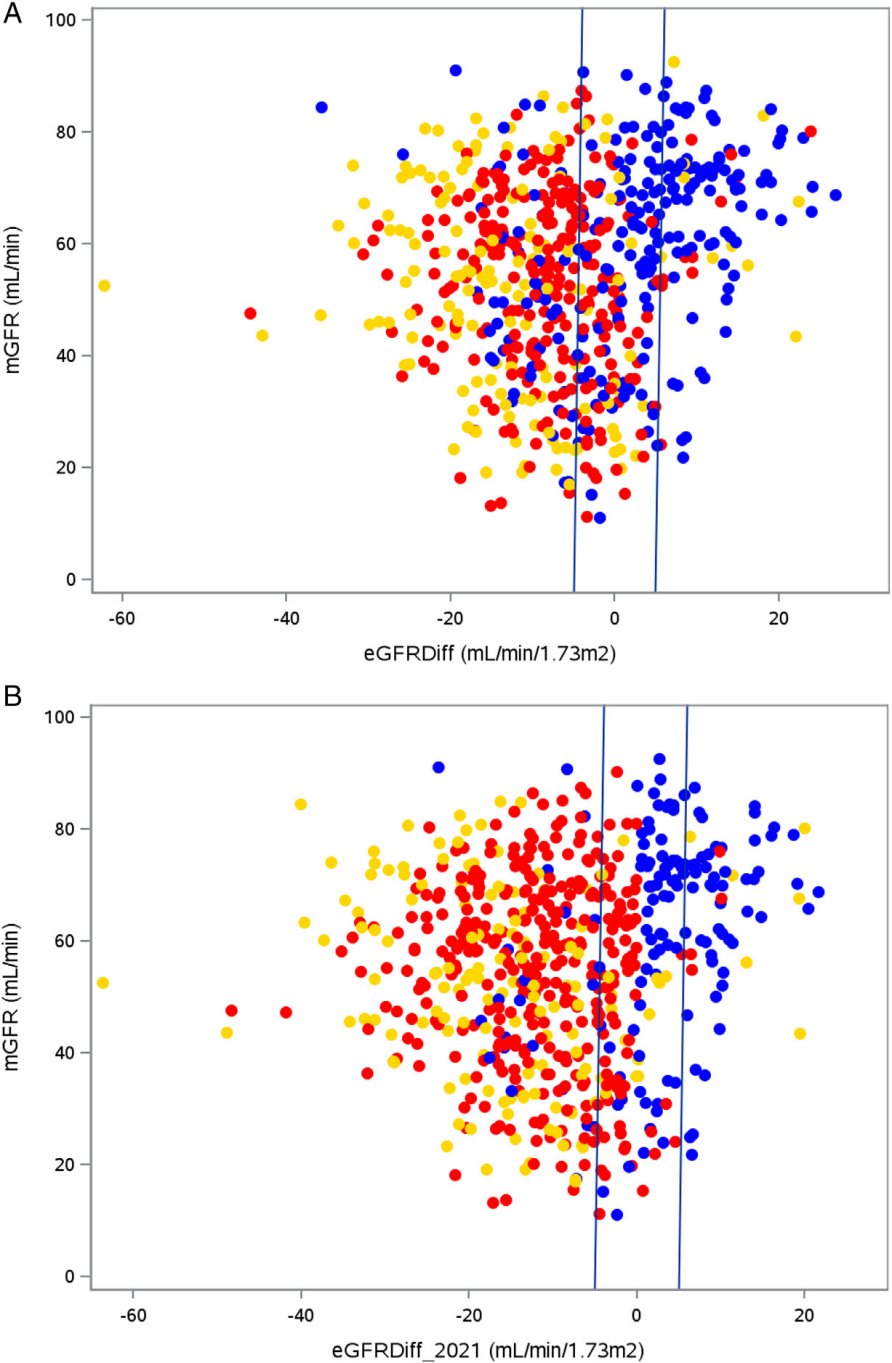
(A) The mGFR by group based on a lower eGFR, showing which estimate between eGFRcr, eGFRcys and eGFRcr-cys is “closest” to the mGFR (ie, which eGFR − mGFR provides the lowest absolute value). The equations used are CKD-EPI 2009/2012. Lower eGFRcys group: those in whom eGFRcys − eGFRcr is <−5 mL/min/1.73 m^2^. Lower eGFRcr group: those in whom eGFRcys − eGFRcr is ≥5 mL/min/1.73 m^2^. X-axis depicts the difference eGFRcys − eGFRcr. Zero is the point where both values are the same. The vertical lines at −5 and +5 separate the 3 groups (lower eGFRcys, reference, and lower eGFRcr groups, respectively). (B) The mGFR by group based on a lower eGFR, showing which estimate between eGFRcr_2021_, eGFRcys_2021_, and eGFRcr-cys_2021_ is “closest” to the mGFR (ie, which eGFR − mGFR provides the lowest absolute value). The equations used are CKD-EPI 2021. Lower eGFRcys_2021_ group: those in whom eGFRcys − eGFRcr is <−5 mL/min/1.73 m^2^. Lower eGFRcr_2021_ group: those in whom eGFRcys − eGFRcr is ≥5 mL/min/1.73 m^2^. X-axis depicts the difference eGFRcys - eGFRcr. Zero is the point where both values are the same. The vertical lines at −5 and +5 separate the 3 groups (lower eGFRcys_2021_, reference_2021_, and lower eGFRcr_2021_ groups, respectively). Abbreviations and definitions: CKD-EPI, Chronic Kidney Disease Epidemiology Collaboration; eGFR, estimated glomerular filtration rate; eGFRcr, creatinine-based estimated glomerular filtration rate; eGFRcr-cys, combined creatinine- and cystatin C–based estimated glomerular filtration rate; eGFRcys, cystatin C–based estimated glomerular filtration rate; eGFRDiff, eGFRcys − eGFRcr; mGFR, measured glomerular filtration rate.

On average, when examining the lower eGFRcys and lower eGFRcr groups, whichever of the 2 eGFR estimates between eGFRcr and eGFRcys was lower was the eGFR estimate that provided lesser bias and higher accuracy when compared to the mGFR gold standard (Table 2). The discrepancy in estimates was relatively large, such that in the lower eGFRcys group, the eGFRcr estimate had a median bias of 11.2 mL/min/1.73 m^2^ and the eGFRcr-cys estimate had a median bias of 3.8 mL/min/1.73 m^2^, whereas the eGFRcys estimate had a median bias of −2.7 mL/min/1.73 m^2^. Conversely, in the lower eGFRcr group, the eGFRcys estimate had a median bias of 13.8 mL/min/1.73 m^2^ and the eGFRcr-cys estimate had a median bias of 9.5 mL/min/1.73 m^2^, whereas the eGFRcr estimate had a median bias of 2.9 mL/min/1.73 m^2^. In the lower eGFRcys group, 94% participants had eGFRcr-cys and 92% participants had eGFRcys estimates within 30% of mGFR. Similarly, in the lower eGFRcr group, 93% participants had eGFRcr estimates within 30% of mGFR. When considering the overall population, the P30 was 91%, 86%, and 79% for the eGFRcr-cys, eGFRcys, and eGFRcr equations, respectively. The combined equation of eGFRcr-cys was the most accurate at the level of the population overall but not the least biased. When considering the lower eGFRcr and lower eGFRcys groups, the lower eGFR between eGFRcr and eGFRcys had a smaller overall bias than the combined equation (Table 2). Fig 1A shows mGFR against eGFRDiff, using a color code to highlight which absolute difference between eGFR and mGFR is the smallest when using either eGFRcr, eGFRcys, or eGFRcr-cys: blue dots represent the participants in whom eGFRcr − mGFR provided the lowest value, red dots represent those in whom eGFRcys − mGFR provided the lowest value, and yellow dots represent those in whom eGFRcr-cys − mGFR was the lowest. In the lower eGFRcr group, 92 (76%) of 121 participants had a lower absolute difference between eGFR and mGFR when using eGFRcr (blue dots), whereas 51.5% participants in the lower eGFRcys group had a lower absolute difference eGFR − mGFR when using eGFRcys (red dots). Those with the lowest absolute difference eGFRcr-cys − mGFR (yellow dots) did not represent the majority of any group (Fig 1A).

**Table 2:** Performance Statistics for the eGFRcr, eGFRcys, and eGFRcr-cys Overall and by Group Based on Which eGFR is Lower Between eGFRcys and eGFRcr (Using CKD-EPI 2009/2012 Equations)

	eGFRcr	eGFRcys	eGFRcr-cys
Overall (n=657)			
P30 (%), mean (95% CI)	79 (76 to 82)	86 (84 to 89)	91 (88 to 93)
Bias (mL/min/1.73 m^2^), median (95% CI)	7.6 (6.8 to 8.9)	1.4 (0.1 to 2.4)	4.7 (4.2 to 5.4)
Lower eGFRcys (eGFRDiff < −5 mL/min/1.73 m^2^) (n=337)			
P30 (%), mean (95% CI)	69 (64 to 74)	92 (89 to 95)	94 (92 to 97)
Bias (mL/min/1.73 m^2^), median (95% CI)	11.2 (10.3 to 12.8)	−2.7 (−3.8 to −1.9)	3.8 (2.8 to 4.7)
Reference (−5 ≤ eGFRDiff < 5 mL/min/1.73 m^2^) (n=199)			
P30 (%), mean (95% CI)	87 (83 to 92)	87 (83 to 92)	87 (83 to 92)
Bias mL/min/1.73 m^2^), median (95% CI)	4.6 (2.7 to 6.3)	4.0 (2.5 to 5.1)	4.3 (2.8 to 5.4)
Lower eGFRcr (eGFRDiff ≥ 5 mL/min/1.73 m^2^) (n=121)			
P30 (%), mean (95% CI)	93 (89 to 98)	68 (59 to 76)	86 (80 to 92)
Bias (mL/min/1.73 m^2^), median (95% CI)	2.9 (0.9 to 4.8)	13.8 (11.4 to 15.6)	9.5 (7.7 to 11.0)

Abbreviations and definitions: CI, confidence interval; CKD-EPI, Chronic Kidney Disease Epidemiology Collaboration; eGFR, estimated glomerular filtration rate; eGFRcr, creatinine-based estimated glomerular filtration rate; eGFRcr-cys, combined creatinine- and cystatin C–based estimated glomerular filtration rate; eGFRcys, cystatin C–based estimated glomerular filtration rate; eGFRDiff = eGFRcys − eGFRcr using the 2012 and 2009 CKD-EPI equations, respectively; P30, percentage of eGFR values within ±30% of mGFR.

The results could be reproduced when using the 2021 CKD-EPI eGFR equations (Fig 1B, Table 3), with the primary difference being that the proportions of participants in the lower eGFRcys, reference, and lower eGFRcr groups were 63%, 26%, and 11%, respectively. In sensitivity analyses, when using the FAS equations, all 4 measures of GFR (eGFRcr_FAS_, eGFRcys_FAS_, eGFRcr-cys_FAS_, and mGFR) were very similar to one another, resulting in considerably lower bias and higher P30 values throughout. The mean P30 was 92%, 92%, and 95% for eGFRcr_FAS_, eGFRcys_FAS_, and eGFRcr-cys_FAS_, respectively (Fig S1, Table S1).

**Table 3:** Performance Statistics for eGFRcr, eGFRcys and eGFRcr-cys overall and by group based on which eGFR is lower between eGFRcys and eGFRcr (using CKD-EPI 2021 equations)

	eGFRcr_2021_	eGFRcys_2021_	eGFRcr-cys_2021_
Overall_2021_ (n=657)			
Overall P30 (%), mean (95% CI)	66 (62 to 69)	86 (84 to 89)	78 (75 to 81)
Bias (mL/min/1.73 m^2^), median (95% CI)	12.0 (10.7 to 12.9)	1.4 (0.1 to 2.4)	9.2 (8.4 to 10.1)
Lower eGFRcys_2021_ (eGFRDiff_2021_ < −5 mL/min/1.73 m^2^) (n=414)			
Overall P30 (%), mean (95% CI)	54 (49 to 59)	92 (90 to 95)	83 (79 to 87)
Bias (mL/min/1.73 m^2^), median (95% CI)	14.5 (13.8 to 15.9)	−1.9 (−2.8 to −1.1)	8.0 (7.1 to 9.1)
Reference_2021_ (−5 ≤ eGFRDiff_2021_ < 5 mL/min/1.73 m^2^) (n=172)			
Overall P30 (%), mean (95% CI)	84 (78 to 89)	84 (78 to 89)	75 (68 to 82)
Bias (mL/min/1.73m^2^), median (95% CI)	6.8 (5.5 to 9.0)	6.2 (4.7 to 8.6)	9.9 (8.5 to 12.4)
Lower eGFRcr_2021_ (eGFRDiff_2021_ ≥ 5 mL/min/1.73 m^2^) (n=71)			
Overall P30 (%), mean (95% CI)	90 (83 to 97)	58 (46 to 70)	59 (47 to 71)
Bias (mL/min/1.73m^2^), median (95% CI)	6.3 (3.1 to 8.6)	16.3 (14.4 to 18.6)	16.7 (13.1 to 19.3)

Abbreviations and definitions: CI, confidence interval; CKD-EPI, Chronic Kidney Disease Epidemiology Collaboration; eGFR, estimated glomerular filtration rate; eGFRcr, creatinine-based estimated glomerular filtration rate; eGFRcr-cys, combined creatinine- and cystatin C–based estimated glomerular filtration rate; eGFRcys, cystatin C–based estimated glomerular filtration rate; eGFRDiff_2021_ = eGFRcys_2021_ – eGFRcr_2021_ using the 2021 CKD-EPI equation for creatinine; P30, percentage of eGFR values within ±30% of mGFR.

## Discussion

In a population of older White adults, we demonstrated that the lower of the 2 eGFRs from cystatin C or creatinine provided the least bias and highest overall accuracy relative to the mGFR. Because cystatin C measurements are used more frequently in clinical practice and because creatinine-based estimates are ubiquitous, clinicians are increasingly faced with discordant eGFR estimates. Although a combined estimate is most accurate at the population level, the eGFRcr and eGFRcys estimates may diverge with varying degrees.^8^^,^^21^ We demonstrated that this divergence holds considerable clinical relevance for death, frailty, and hospitalizations,^15^^,^^16^ even after accounting for kidney function. Moreover, among those with discrepancy, clinicians naturally wonder which may be a more accurate estimate of mGFR. If confirmed, our findings will have important clinical implications for the diagnosis of chronic kidney disease, defining prognosis, and particularly for drug dosing in older patients, in whom chronic kidney disease prevalence, polypharmacy, and risk of adverse drug events are all high.

Currently, estimation of kidney function on the basis of serum creatinine^3^^,^^24^ remains the clinical standard of care to decide on medication dosing. However, there is growing evidence that cystatin C could provide a better assessment of kidney function. A recent systematic review^25^ found that eGFRcys was more predictive of drug levels and drug clearance than eGFRcr. Although our study did not evaluate medication dosing specifically, our findings suggest that the lower of the 2 estimates, between eGFRcr and eGFRcys, may be more accurate. Importantly, half of our population had a lower eGFRcys, highlighting the importance of its measurements in older persons, such as those studied in this study, and highlighting the degree of GFR estimation inaccuracy that may be prevalent in clinical care for older adults without assessment of cystatin C.

Our prior research evaluating persons with lower eGFRcys than eGFRcr demonstrates that such persons have lower muscle mass,^26^ greater degree of comorbidities, and lower functional status.^15^^,^^16^ Therefore, a priori, we hypothesized that eGFRcys would provide a more accurate estimate of mGFR among those in the lower eGFRcys group. We confirmed this hypothesis in our study. Not only was this the case relative to eGFRcr, but eGFRcys was also more accurate than the 2021 combined equation within the lower eGFRcys group. On the other hand, we also found that eGFRcr was a more accurate estimate of mGFR among those with lower eGFR by creatinine. eGFRcys did not outperform eGFRcr in the lower eGFRcr group. One reason for this could be the non-GFR determinants of cystatin C (which have not yet been as thoroughly investigated as the non-GFR determinants of creatinine). Although finding an eGFRcr lower than eGFRcys was less common in this cohort of older adults (32%), in such individuals, eGFRcr provided the best estimate of mGFR, and the measurement of eGFRcys could be misleading if it served as the estimate used for clinical decision making. This study provides a new tool to decide which of the eGFR estimates may be more reliable, finding that the estimate that is lower is a more accurate estimate, on average. It also highlights the clinical importance of obtaining both estimates of GFR in older adults.

In this study, eGFR was calculated using the CKD-EPI equations from 2009 and 2012, and the pattern of results was similar when using the new 2021 CKD-EPI equations. Because our study population was 100% White, using the new equations without a race coefficient only shifted the values of eGFRcr upward. Indeed, because the eGFRcys equation remained the same (ie, that of 2012), the upward shift in eGFRcr using the 2021 CKD-EPI equation artifactually pushed more participants into the lower eGFRcys group. It has been shown that the new equation (2021) without a race factor is less accurate than the “old” CKD-EPI equations (2009/2012) in White Europeans.^21^^,^^27^^,^^28^ Indeed, the P30 for creatinine in participants from the lower eGFRcys group was only 54% when using the CKD-EPI 2021 equation compared with the P30 of 69% when using the CKD-EPI 2012 equation. We also evaluated the FAS equations but opted against using them in our main analysis because they were originally derived in BIS and, therefore, fit overly well in our study population. Nonetheless, using the FAS equations in supplemental analyses showed similar results to those in our main analyses.

The strengths of our study include the large sample size for a study providing 3 measures of kidney function, including mGFR. Its evaluation in an older population is another key strength because discrepant eGFR estimates are common in this age group, as are the clinical risks of inaccurate GFR estimation and adverse drug events. The study also has an important limitation. The study evaluated eGFR performance in an older community-dwelling German population, 100% of whom were White. Generalizability of our findings to younger and more diverse populations will require future study.

In conclusion, in this population of older White adults, the lower of the 2 eGFR estimates derived from eGFRcys versus eGFRcr provides a less biased and more accurate estimate of mGFR when comparing groups on the basis of eGFRDiff. The combined equation of eGFRcr-cys was most accurate (although not the least biased) at the population level. This remained true whether eGFR was calculated using the CKD-EPI equations from 2009/2012 or 2021. Our findings are relevant to older adults and will have to be replicated and validated in future studies and confirmed in other populations. Guidelines already suggest the use of cystatin C, and our findings support its use in older populations because it may be the more accurate estimate of mGFR in those who have a lower eGFR by this estimate.
