# Supplementary material for: Estimated GFR Accuracy When Cystatin C– and Creatinine-Based Estimates Are Discrepant in Older Adults
Source: Kidney Med. 2023 Mar 13;5(5):100628. doi: 10.1016/j.xkme.2023.100628 (PMC10165149; doi:10.1016/j.xkme.2023.100628)
Supplement: Supplementary File (PDF) — Figure S1; Table S1. [file mmc1.pdf]

Figure S1. mGFR by group based on lower eGFR, showing which estimate between $eGFRcr_{FAS}$, $eGFRcys_{FAS}$, and $eGFRcr\text{-}cys_{FAS}$ is "closest" to mGFR (ie which eGFR – mGFR provides the lowest absolute value). The equations used are FAS.

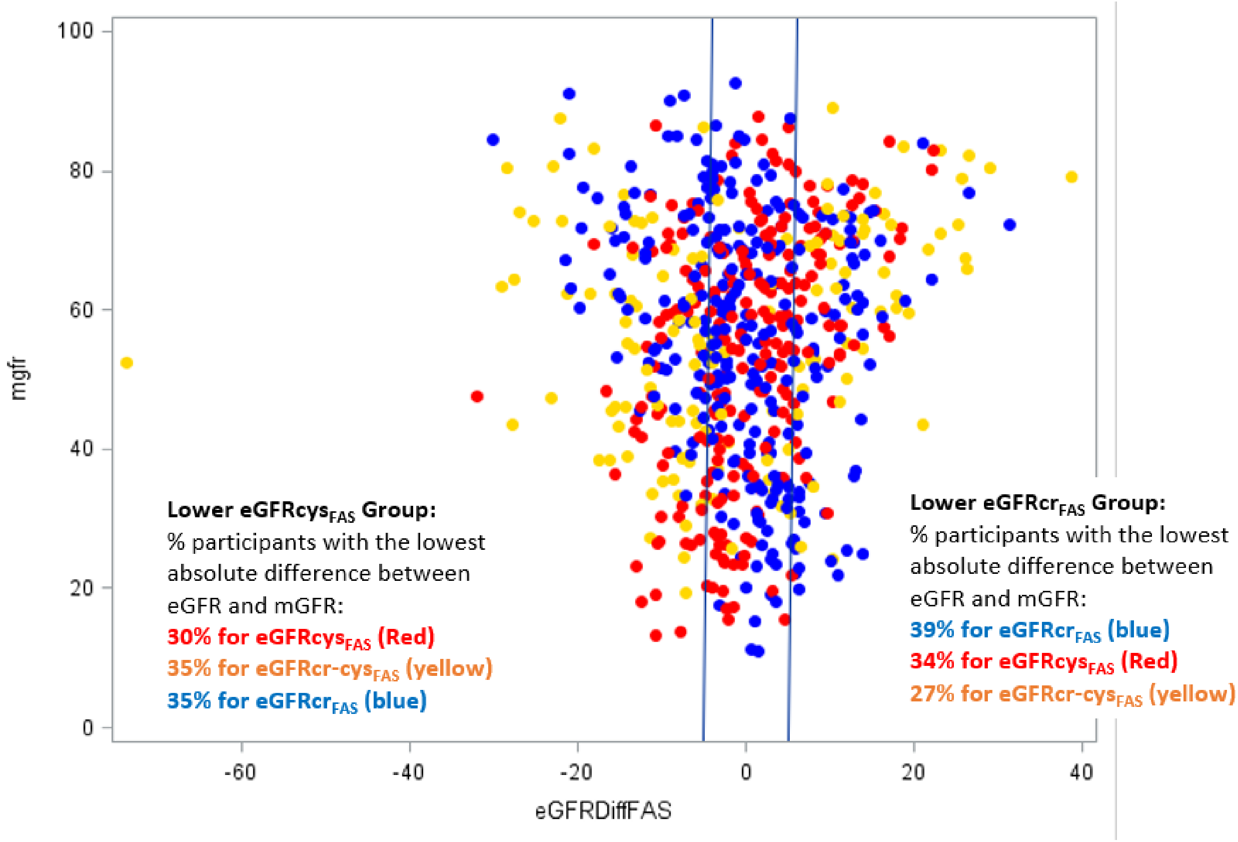

Lower $eGFRcys_{FAS}$ group: those in whom eGFRcys – eGFRcr < -5 mL/min/1.73m^2
Lower $eGFRcr_{FAS}$ group: those in whom eGFRcys – eGFRcr ≥ 5 mL/min/1.73m^{2}X axis depicts the difference eGFRcys – eGFRcr. Zero is the point where both values are the same. The vertical lines at -5 and +5 separate the 3 groups (Lower eGFRcys, reference, and Lower eGFRcr groups respectively)

Table S1. Performance statistics for the $eGFRcr_{FAS}$, $eGFRcys_{FAS}$ and $eGFRcr\text{-}cys_{FAS}$ by group (using FAS equations)

	**$Overall_{FAS}$ (n=657)**		
	**$eGFRcr_{FAS}$**	**$eGFRcys_{FAS}$**	**$eGFRcr\text{-}cys_{FAS}$**
Average P30 (95% CI) %	92% (90%; 94%)	92% (90%; 94%)	95% (94%; 97%)
Median bias (95% CI) mL/min/1.73m^2	0.5 (-0.4; 1.3)	-0.2 (-0.9; 0.5)	0.1 (-0.7; 0.6)
	**Lower $eGFRcys_{FAS}$ ($eGFRDiff_{FAS}$ < -5 mL/min/1.73m^2) (n=187)**		
	**$eGFRcr_{FAS}$**	**$eGFRcys_{FAS}$**	**$eGFRcr\text{-}cys_{FAS}$**
Average P30 (95% CI) %	87% (82%; 92%)	93% (89%; 97%)	96% (93%; 99%)
Median bias (95% CI) mL/min/1.73m^2	5.1 (4.1; 6.6)	-5.5 (-7.4; -4.2)	-0.8 (-1.9; 0.4)
	**$Reference_{FAS}$ (-5 ≤ $eGFRDiff_{FAS}$ < 5 mL/min/1.73m^2) (n=284)**		
	**$eGFRcr_{FAS}$**	**$eGFRcys_{FAS}$**	**$eGFRcr\text{-}cys_{FAS}$**
Average P30 (95% CI) %	94% (91%; 97%)	96% (94%; 98%)	96% (94%; 98%)
Median bias (95% CI) mL/min/1.73m^2	0.5 (-1.0; 1.3)	-0.2 (-0.7; 0.8)	0.2 (-1.0; 1.0)
	**Lower $eGFRcr_{FAS}$ ($eGFRDiff_{FAS}$ ≥ 5 mL/min/1.73m^2) (n=186)**		
	**$eGFRcr_{FAS}$**	**$eGFRcys_{FAS}$**	**$eGFRcr\text{-}cys_{FAS}$**
Average P30 (95% CI) %	95% (92%; 98%)	86% (81%; 91%)	94% (90%; 97%)
Median bias (95% CI) mL/min/1.73m^2	-4.6 (-5.7; -3.4)	6.7 (5.0; 8.0)	0.9 (-0.8; 2.0)

CI: confidence interval

$eGFRDiff_{FAS}$ = eGFRcys – eGFRcr using the FAS eGFR equations
